# Supplementary material for: Recruiting strategic human capital from MNCs—Does hiring MNC managers enable exporting in domestic firms?
Source: PLoS One. 2021 Oct 7;16(10):e0257922. doi: 10.1371/journal.pone.0257922 (PMC8496813; doi:10.1371/journal.pone.0257922)
Supplement: S1 Appendix — (DOCX) [file pone.0257922.s001.docx]

# **APPENDIX**

**TABLE A1** Balancing results for means

|  | Diff: mean (before balancing) | Diff: mean (after balancing) |
| --- | --- | --- |
| Young firm (d) | 0.15 | 0.00 |
| International diversity | 0.05 | 0.00 |
| Hierarchical specialization | 0.91 | 0.00 |
| Log employees | 1.10 | 0.00 |
| Hiring ratio | 0.70 | 0.00 |
| Relative firm wage | 1.64 | 0.00 |
| Capital intensity | -7628.00 | -6.00 |
| Investment ratio turnover | -11.98 | 0.00 |
| Share managers and professionals | 0.03 | 0.00 |
| Share with non-MNC management experience | 0.00 | 0.00 |
| Share tertiary employees | 0.03 | 0.00 |
| Log productivity | 100684.00 | 6.00 |
| Education dummy 1 | -0.04 | 0.00 |
| Education dummy 2 | -0.01 | 0.00 |
| Education dummy 3 | 0.00 | 0.00 |
| Education dummy 4 | 0.02 | 0.00 |
| Education dummy 5 | 0.06 | 0.00 |
| Education dummy 6 | -0.03 | 0.00 |
| Education dummy 7 | -0.01 | 0.00 |
| Education dummy 8 | 0.00 | 0.00 |
| Education dummy 9 | 0.00 | 0.00 |
| NACE 1 | 0.00 | 0.00 |
| NACE 2 | 0.01 | 0.00 |
| NACE 3 | 0.01 | 0.00 |
| NACE 4 | 0.04 | 0.00 |
| NACE 5 | 0.02 | 0.00 |
| NACE 6 | 0.02 | 0.00 |
| NACE 7 | 0.04 | 0.00 |
| NACE 8 | 0.01 | 0.00 |
| NACE 9 | -0.06 | 0.00 |
| 2011 | -0.03 | 0.00 |
| 2012 | -0.01 | 0.00 |
| 2013 | 0.01 | 0.00 |
| 2014 | 0.04 | 0.00 |
| 2015 | 0.04 | 0.00 |

**TABLE A2** Robustness checks with IV regression, (dependent var: export status)

|  | (1) |
| --- | --- |
|  | Dummy: exporter |
| Hiring MNC manager | 0.0170 |
|  | (0.001) |
| Young firm | -0.0053 |
|  | (0.000) |
| International diversity | 0.0021 |
|  | (0.023) |
| Hierarchical specialization | 0.0040 |
|  | (0.000) |
| Dummy: Domestic exporter background | 0.0060 |
|  | (0.000) |
| Log employees | 0.0030 |
|  | (0.000) |
| Hiring ratio | -0.0002 |
|  | (0.000) |
| Capital intensity | 0.0000 |
|  | (0.885) |
| Share managers and professionals | -0.0021 |
|  | (0.001) |
| Investment share turnover | -0.0000 |
|  | (0.535) |
| Share with non-MNC management experience | 0.0038 |
|  | (0.000) |
| Share tertiary employees | 0.0022 |
|  | (0.000) |
| Lagged labor productivity | 0.0000 |
|  | (0.004) |
| Constant | -0.0009 |
|  | (0.220) |
| Year dummies | Yes |
| Sector dummies | Yes |
| Education field dummies | Yes |
| Observations | 1923620 |
| #Firms | 474926 |
| *R*^2^ | 0.014 |

*p*-values in parentheses
