# Supplementary material for: Recruiting strategic human capital from MNCs—Does hiring MNC managers enable exporting in domestic firms?
Source: PLoS One. 2021 Oct 7;16(10):e0257922. doi: 10.1371/journal.pone.0257922 (PMC8496813; doi:10.1371/journal.pone.0257922)
Supplement: S1 File — (DOCX) [file pone.0257922.s002.docx]

**Data sharing:**

**Description of the data set and the third-party source:**

The data structure underlying the publication is a huge interlinked national registry dataset covering information on Swedish firms and the Swedish population over time. It can be understood as a rich linked employer-employee database, which allows for panel coverage.

The data underlying the publication is owned by the Swedish Statistical Office (SCB) and hosted in the MONA-system. The MONA system is a remote access data infrastructure, from which only aggregated results (tables, regressions, graphs etc.) but no unprocessed raw data can be extracted. Publicly sharing a minimal dataset is therefore legally not possible. However, access to MONA is principally possible for research organizations. Obtaining access requires application because the information contained in MONA pertains also to sensitive individual data requiring personalized access to be able track and control data treatment.

**How to get access:**

SCB grants automatic full data access to employees of organizations, who already have access to MONA. If the employee's organization has no access to MONA, applications can be directed to the MONA-group in SCB ([mona@scb.de](mailto:mona@scb.de)). The application process entails costs and therefore may not be recommendable for the sole purpose of a replication.

For replication purposes, we encourage requesting (temporary) membership at the corresponding author's home organization CIRCLE - Centre for Innovation Research. Affiliated members will the full rights to access the CIRCLE-data-infrastructure including the full raw data underlying this publication. Request for (temporary) affiliated membership should be addressed to Markus Grillitsch, [markus.grillitsch@keg.lu.se](mailto:markus.grillitsch@keg.lu.se). Please copy the corresponding author of this publication Torben Schubert, [torben.schubert@circle.lu.se](mailto:torben.schubert@circle.lu.se), who will make sure to treat the process efficiently.
